# Restorative effects of *Lactobacillus rhamnosus LR-32* on the gut microbiota, barrier integrity, and 5-HT metabolism in reducing feather-pecking behavior in laying hens with antibiotic-induced dysbiosis

**DOI:** 10.3389/fmicb.2023.1173804

**Published:** 2023-04-26

**Authors:** Chenxuan Huang, Qiaoxian Yue, Li Sun, Keqian Di, Duanli Yang, Erying Hao, Dehe Wang, Yifan Chen, Lei Shi, Rongyan Zhou, Guoxian Zhao, Hui Chen

**Affiliations:** ^1^College of Animal Science and Technology, Hebei Agricultural University, Baoding, China; ^2^Department of Animal Nutrition and Management, Swedish University of Agricultural Science, Uppsala, Sweden; ^3^Department of Animal Breeding and Genetics, Swedish University of Agricultural Sciences, Uppsala, Sweden; ^4^School of Basic Medical Sciences, Hebei University, Baoding, Hebei, China

**Keywords:** intestinal dysbacteriosis, microbiome-gut-brain axis, feather-pecking behavior, probiotics, laying hen

## Abstract

The development of abnormal feather-pecking (FP) behavior, where laying hens display harmful pecks in conspecifics, is multifactorial and has been linked to the microbiota-gut-brain axis. Antibiotics affect the gut microbial composition, leading to gut-brain axis imbalance and behavior and physiology changes in many species. However, it is not clear whether intestinal dysbacteriosis can induce the development of damaging behavior, such as FP. The restorative effects of *Lactobacillus rhamnosus LR-32* against intestinal dysbacteriosis-induced alternations need to be determined either. The current investigation aimed to induce intestinal dysbacteriosis in laying hens by supplementing their diet with the antibiotic lincomycin hydrochloride. The study revealed that antibiotic exposure resulted in decreased egg production performance and an increased tendency toward severe feather-pecking (SFP) behavior in laying hens. Moreover, intestinal and blood-brain barrier functions were impaired, and 5-HT metabolism was inhibited. However, treatment with *Lactobacillus rhamnosus LR-32* following antibiotic exposure significantly alleviated the decline in egg production performance and reduced SFP behavior. *Lactobacillus rhamnosus LR-32* supplementation restored the profile of the gut microbial community, and showed a strong positive effect by increasing the expression of tight junction proteins in the ileum and hypothalamus and promoting the expression of genes related to central 5-HT metabolism. The correlation analysis revealed that probiotic-enhanced bacteria were positively correlated, and probiotic-reduced bacteria were negatively correlated with tight junction-related gene expression, and 5-HT metabolism, and butyric acid levels. Overall, our findings indicate that dietary supplementation with *Lactobacillus rhamnosus LR-32* can reduce antibiotic-induced FP in laying hens and is a promising treatment to improve the welfare of domestic birds.

## Introduction

The gut microbiota consists of a complex and relatively stable community of microorganisms widely reported to influence metabolism, the immune and endocrine systems, and neuromodulation in the host (Cryan and Dinan, 2012; Chen et al., 2021). However, it can be rapidly altered by a number of exogenous and endogenous factors, such as antibiotic therapy, inflammatory disease, diet, or stress (Borre et al., 2014; Schokker et al., 2017). Gut microbial disorders can disrupt intestinal-brain homeostasis and tight junctions in these systems. The resultant increase in intestinal permeability and formation of a “leaky gut,” can increase the infiltration of large amounts of pathogenic bacteria and toxic metabolites into the bloodstream, causing local or systemic inflammation. The inflammatory response can disrupt the blood-brain barrier (BBB), known as the leaky brain, and substances that promote inflammation, such as lipopolysaccharides, may enter the brain, leading to neuroinflammation, mental disorders, and abnormal behavior (Obrenovich, 2018; Yousefi et al., 2022). On the other hand, probiotics have shown beneficial effects on host health and behavior, including reversing stress or antibiotic-induced gut microbial disorders and restoring physiological and behavioral changes in the host by modulating gut-brain axis signaling via hormones, immune factors, etc., (Arslanova et al., 2021; Mindus et al., 2021; Wang et al., 2021; Huang et al., 2022).

Feather-pecking (FP) is typically an abnormal behavior in layer flocks, causing increased social stress, feather loss, skin injury, and other pecking habits, and can escalate into cannibalism in severe cases; seriously affecting animal welfare and health (Bestman et al., 2017). Previously, this behavior has been proposed to be a consequence of dysregulation of the gut-brain axis in laying hens. Disruptions in the microbiome of the gut (van der Eijk et al., 2019a), the immune system (van der Eijk et al., 2019b), the monoamine neurotransmitter systems that regulate behavior (Kops et al., 2013) and a hyperactive Hypothalamic-Pituitary-Adrenal (HPA) axis (Kjaer and Guémené, 2009) have been observed in FP birds. This evidence suggests that the microbiota-gut-brain axis plays a vital role in the development of FP behavior. For instance, Bolhuis et al. (2009) reported that FP birds showed lower whole blood 5-HT levels and higher feather-pecking frequency than non-FP birds. In addition, there was evidence of higher Clostridium abundance and lower *Lactobacillus* abundance in the feces of FP birds compared to non-FP birds (Birkl et al., 2018). Differences in the levels of gut microbiota-derived metabolites, including short-chain fatty acids between FP and non-FP birds have also been reported (Meyer et al., 2013). Furthermore, our previous study identified pecked feather phenotypes with different gut microbiota, metabolite profiles, and 5-HT metabolism (unpublished data). Whether altering the gut microbiota contributes to FP symptoms or underly the development of FP, however, remains unknown.

Hence, we hypothesized that antibiotic-induced dysbiosis in chickens could provoke injurious behavior. Experimental chickens were exposed to lincomycin hydrochloride to disrupt the gut microbial community, and the effects of *Lactobacillus rhamnosus LR-32* as a potential intervention against antibiotic-induced feather-pecking behavior were evaluated.

## Materials and methods

### Antibiotic and probiotic

The antibiotic Lincomycin (Lincomycin hydrochloride sodium salt, CAS Number: 859-18-7, Sigma-Aldrich, Shanghai Warehouse, China) was used in this study. The probiotic is a single strain of *Lactobacillus rhamnosus LR-32* (catalog number MF-009807, DuPont, Wilmington, DE, USA) with a minimum of 1 × 10^9^ CFU living bacteria count and stored at 4°C.

### Birds, housing, and experiments

A total of 216 Hy-Line Brown pullets at 70 weeks of age were acquired from a commercial breeding farm and housed until 88 weeks of age in an animal husbandry teaching experimental base at Hebei Agricultural University until 88 weeks of age. The pullets were kept in a room (10.5 m × 5.8 m x 3.5 m, L × W × H) equipped with 36 cages (100 cm × 70 cm × 55 cm, L × W × H), each providing separate feeding troughs and nipple drinkers. Birds were housed six per cage, provided with perches and were fed 2 times per day at 06:00 and 14:00, access to food and water *ad libitum*. The composition and nutritional levels of the basal diet are detailed in Table 1. Light was provided between 06:00 and 16:00 h daily and the average temperature was maintained at 16–23°C.

**TABLE 1:** Ingredients and chemical composition of basal diet.

Ingredients	Content, %
Corn	62.20
Soybean meal	21.90
Wheat bran	1.50
CaHPO_4_	1.60
Soybean oil	1.50
Lys	0.07
Fine powder	3.30
NaCl	0.20
NaHCO_3_	0.20
Midding flour	7.30
Trace elements^1^	0.20
Multivitamin^2^	0.03
Total	100.00
**Nutrient subtilis^3^**	
ME/(MC/kg)	2.735
CP	15.317
Ca	4.106
TP	0.482
AP	0.379
CF	4.096
Na	0.168
Lys	0.715
Met	0.336
Trp	0.164

^1^The trace elements provided the following per kg of diet: Cu (as copper sulfate), 0.8 g, Fe (as ferrous sulfate), 6 g, Mn (as manganese sulfate), 9 g, Zn (as zinc sulfate), 6 g, I, 90 mg, and Se (as sodium selenite), 21 mg.

^2^The multivitamin provided the following per kg of diets: vitamin A, 330,000 IU, vitamin B1, 20 mg, vitamin B2, 500 mg, D-pantothenate calcium, 1,200 mg, vitamin B6, 300 mg, vitamin D3, 82,500 IU, vitamin E, 2,000 IU, vitamin K, 180 mg, biotin, 14 mg, folic acid, 55 mg, niacin, 2,300 mg, and choline, 45 g.

^3^The nutrient levels were calculated.

After a 2-week adaptation phase, hens were randomly divided into four groups (*n* = 54 per group) in a completely randomized design. Each group consisted of 3 replicates with 18 hens per replicate, and treated as follows: group A (control group: basic diet for 4 weeks), group B (antibiotics group: basic diet with lincomycin for 4 weeks), group C (natural restoration group: basic diet with lincomycin for 4 weeks followed by basic diet without lincomycin for 8 weeks), Group D (probiotics group: basic diet with lincomycin for 4 weeks followed by basic diet with *Lactobacillus rhamnosus LR-32* for 8 weeks). The antibiotic dosage was 50 mg/kg diet according to the standard of the National Veterinary Drug Safety Agency (Papich, 2016), and the *Lactobacillus rhamnosus LR-32* was used at 1 × 10^10^ CFU/kg diet.

**FIGURE 1:**
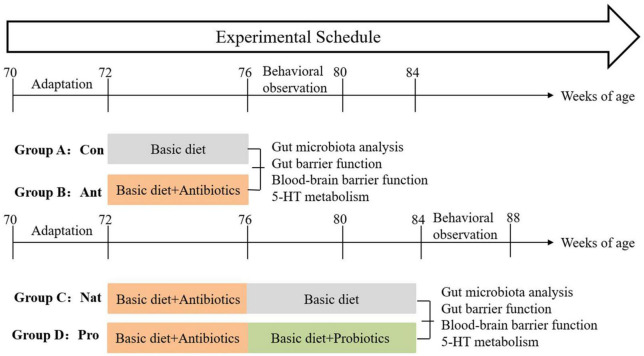
Schematic diagram showing treatment groups and the experimental timeline.

To prevent cross-contamination of diets and behaviors such as feather-pecking, wooden boards were placed between each group’s troughs and cage walls to ensure that the hens were not visible to each other and remained relatively independent. All hens were subjected to production performance measurement and 2 weeks of behavioral observation. Subsequently, seven hens were randomly selected from each treatment group for further analysis of gut microbiota, gut-barrier function, blood-brain barrier function, and 5-HT metabolism in the gut-brain axis. The experimental procedure is illustrated in Figure 1.

All the following experimental procedures were approved by the Animal Ethics Committee of Hebei Agricultural University (Permit Number: HB/2021/03), including animal use and welfare.

### Production performance

The number and weight of eggs, as well as the amount of feed added to each cage were recorded daily for each group. The weekly food consumption was used to calculate the average daily feed intake (ADFI) using formular *ADFI* = (cumulated weekly consumption/7 days)/bird number. Feed conversion ratio (FCR) was calculated as ratio of the total amount of feed consumed per egg produced normalized to the total grams of eggs produced.

### Behavioral observation

Behavioral observations were performed via video recordings with two hanging video cameras (Sony/DSC-HX400, Japan), which allowed two cages from different groups to be recorded at the same time, e.g., A and B or C and D. Recordings were made twice daily (90 min at 9:00 h and 90 min at 14:00 h) over 2 weeks, and 9 cages per group were recorded. Hens were tagged with different colored ankle rings to facilitate differentiation. The video recordings were analyzed by software (HyperSnap-DX, Murrysville, PA, USA) and the recorded variables were gentle and severe feather-pecking.

Gentle feather-pecking (GFP) and severe feather pecking (SFP) was defined as described previously (Daigle et al., 2015). GFP is characterized by non-aggressive gentle pecks lasting more than 4 s at the tips and edges of the feather of another bird without feather removal, and is normally ignored by the recipient. On the hen other hand, SFP involves using forceful beak pecks that remove feathers or cause injury and is often responded to by the recipient with avoidance or retaliation. Behavioral observations were carried out by an experienced observer who was blinded to the treatments.

After the behavioral observation, all the birds were weighed and scored for feather coverage on the head, back, wings, and tail using a 4-point photographic scale as previously described (Kjaer et al., 2005), and the average feather score = is the sum of feather scores for all parts divided by 4.

### Sample collection and preparation

Seven hens from each group were randomly selected for blood collection via the sub-wing vein. Blood samples were centrifuged at 3,000 rpm for 15 min, and the serum was separated and stored at −20°C for analysis. Immediately following euthanasia, the ileum, cecum content, and brain (the whole hypothalamus was dissected using a stereotaxic atlas) were carefully removed under sterile conditions and frozen in liquid nitrogen before storage at −80°C for later analysis.

### Gut microbiota profiling

Total genomic bacterial DNA was extracted using the Power Soil^®^ DNA Isolation kit (Mobio, Carlsbad, CA, USA) according to the manufacturer’s instructions. The primers correspond to the region: 16S V3-V4 region primer 338F (ACTCCTACGGGAGGCAGCA) and 806R (GGACTACHVGGGTWTCTAAT). PCR amplification was carried out in a 25 μl reaction system and the condition of PCR amplification was: initial pre-denaturation at 94°C for 4 min, denaturation at 98°C for 10 s, renaturation at 58°C for 30 s, elongation at 72°C for 2 min, 30 cycles, and then the last elongation step at 72°C for 10 min. After PCR amplicons were purified and quantified, then were sequenced on the Illumina HiSeq PE250 platform (Illumina, San Diego, CA, USA) at the RealBio Technology Inc. (Shanghai, China).

The 16S rRNA gene sequencing data processing was performed using the Quantitative Insights into Microbial Ecology (QIIME, v1.8.0) pipeline. To obtain high -quality sequences, the Illumina raw reads were subjected to quality control measures, including low-quality and length filtering. Operational taxonomic units (OTUs) were created using an identity threshold of >97% for each high-quality sequence. The RDP classifier (version 2.2)^1^ was used to assign taxonomy to each OTU based on the 16S rRNA database.^2^ Alpha diversity indices (including Chao1, Observed species, Shannon index and Simpson index) were calculated to assess microbial species homogeneity. The beta diversity was assessed through the QIIME pipeline^3^ by applying weighted UniFrac distances. The linear discriminant analysis (LDA) effect size method was performed to identify the specific taxa in the cecal microbiota that were significantly associated with antibiotic or probiotic treatment [LDA score (log 10) > 2]. Bar chart comparing the differences in the relative abundance of different taxonomic units at the phylum level after antibiotic or probiotic treatment. Finally, PICRUSt was used to predict the functions of gut microbiota and annotated in the KEGG database. The differences in the predicted function of cecal microorganisms after antibiotic or probiotic treatment were compared by STAMP (Parks et al., 2014).

### Gut metabolic profiling

Cecum samples from each group were used to detect gut-microbial metabolism-derived short-chain fatty acids (SCFAs). Thawed samples were extracted with 300 μL of methanol, the proteins precipitated, and the supernatant retained for liquid chromatography/mass spectrometry (LC/MS) analysis. 20 μL from each sample was pooled to form the QC sample. Supernatants (200 μL) were analyzed on a UHPLC-QTOF-MS system to determine the SCFAs type and concentration. Metabolites were assigned using the Chenomx NMR Suite 8.2 software (Chenomx Inc., Edmonton, AB, Canada).

### Biochemical detection in serum

Enzyme-linked immunosorbent assays (ELISA) were used to analyze biochemical indicators in the serum. The concentrations of tryptophan (Trp), serotonin (5-HT), 5-hydroxy indole acetic acid (5-HIAA), diamine oxidase (DAO), lipopolysaccharide (LPS), tumor necrosis factor-α (TNF-α), and interleukin-6 (IL-6) concentration were measured with commercial ELISA kits (Shanghai Jianglai Biotechnology Co., Ltd., Shanghai, China) according to the instructions of the manufacturer. The limits of detection and quantification, assay sensitivity, and inter and intra-assay coefficient of variation were as follows, respectively: Trp (5.46–350 μmol/L, 1.0 μmol/L, 10%, 15%), 5-HT (7.5–240 ng/mL, 1.0 ng/mL, 10%, 15%), 5-HIAA (2.5–160 ng/mL, 1.0 ng/mL, 10%, 10%), LPS (0.156–10 ng/mL, 0.076 ng/mL, 10%, 15%), DAO (3.12–200 ng/mL, 1.5 ng/mL, 10%, 15%), TNF-α (3.12–200 pg/mL, 1.4 pg/ml, 10, 10%), and IL-6 (3.12–200 pg/mL, 1.52 pg/mL, 10%, 10%).

### Western blotting

Protein expressions in the ileum and hypothalamic tissues were determined by Western blotting as previously described (Hao et al., 2020). In short, protein extraction was performed by using RIPA lysis buffer, and protein concentration was detected by the bicinchoninic acid (BCA) method. Proteins (90 μg) were separated by SDS-PAGE electrophoresis, transferred on polyvinylidene fluoride (PVDF) membrane, and incubated with primary antibody against ZO-1 (Invitrogen, 33-9100), claudin-1 (Invitrogen, 51-9000), occludin (Invitrogen, 71-1500), and actin (ABclonal, AC026); all at 1:1,000. Protein expressions were revealed with appropriate secondary antibodies (1:5,000; Abcam, ab6721).

### RNA extraction and real-time PCR analysis

Tissue RNA was extracted using the total RNA extraction kit (12183-555, Invitrogen, Waltham, MA, USA) following the manufacturer’s instructions. RNA quality was verified with the SmartSpec Plus spectrophotometer (BIO-RAD). Total RNA was reverse transcribed with the Super Script™ III First-Strand Synthesis kit (11752-050, Invitrogen, Waltham, MA, USA) and the qPCR performed with the Thermo Scientific™ DyNAmo HS SYBR Green qPCR Kit (10788298, Invitrogen, Waltham, MA, USA). The primer sequences for ZO-1, claudin-1, occludin, TLR4, TNF-α, 5-HTR1A, TPH2, and MAO-A are listed in Table 2. β-actin was used as the internal reference gene and the relative expression level of target genes were calculated by the 2^–Δ^ ^Δ^ ^Ct^ method.

**TABLE 2:** Primer sequences used for real-time PCR.

Gene	GenBank accession	Primer sequences (5′to3′)	Size (bp)
*ZO-1*	XM_046925214.1	CCTGCCAGCCATCATTCTGA TTGGCTCATAGCGCTTGTCA	136
*Claudin-1*	NM_001013611.2	GGTGTACGACTCGCTGCTTA	124
		CTTCATGCACTTCATGCCCG	
*Occludin*	NM_205128.1	TCTGGGAAGGGCTGAGGTC	171
		ATGCCTTCCCAAAAAGCCCT	
*TPH2*	NM_001001301.2	TGACATCACGTGACATGGCT TGTGTTGAGGAATCGCTGGT	179
*MAO-A*	NM_001030799.1	ATGAAGAGAAGAACTGGACTATGGA CAGAAGGTGGTGGTGAATGGT	298
*5-HTR1A*	NM_001170528.1	TCACGGACCCCATCGACTAT	143
		GTCAGGATTTGAGCGGTCCT	
*TNF*-α	MF000729.1	GGTGCGGCCATATAAGCG TCAATTGACGTCGTTCTGAGC	168
*TLR4*	NM_001030693.2	ACCTCAATGCGATGCACTCT AGTCCGTTCTGAAATGCCGT	112
β*-actin*	NM_205518.1	CGGACTGTTACCAACACCCA TCCTGAGTCAAGCGCCAAAA	115

### Statistical analysis

Pecking behaviors within each 30-min block were grouped for analysis. Since the number of pecking behavior per hen followed a Poisson distribution, the behavioral data were analyzed using a binary scale, i.e., with or without pecking behavior, during flora disorder or flora recovery.

All statistical analyses were performed with the SPSS software (version 22.0, IBM SPSS, Chicago, IL, USA). Production performance, physiological, and plumage scores were analyzed by independent two-sample *t*-test. All bar graphs (alpha and beta diversity indexes, microbiota, metabolite, protein abundances, and gene expression data) were generated with GraphPad Prism 8.0 (GraphPad Software, Chicago, IL, USA), R packages (v3.2.0), and Chenomx NMR Suite 8.2 (Edmonton, AB, Canada). The correlational analyses among the SFP behavior, physiological indicators, and the relative abundance of gut microbiota were performed using the built-in function cor of the R package. Comparative analyses were performed with unpaired Students *t*-test, Mann–Whitney U-test, and Kruskal–Wallis test. The results were presented as mean ± SD. A *p*-value of less than 0.05 (*p-value < 0.05) and (**p-value < 0.01) was considered statistically significant. The false discovery rate (FDR) correction was applied to control multiple hypothesis testing errors.

## Results

### Production performance and feather scoring

The effect of different dietary treatments on the egg production performance and feather condition of laying hens are shown in Table 3. After 4 weeks of treatment with antibiotics, egg production was significantly decreased (*P* = 0.037), while a trend (*P* = 0.086) for increased FCR was observed in the antibiotic (Ant) group compared with those in the control (Con) group. The administration of probiotics (Pro) treatment reversed the decrease in egg production induced by antibiotic treatment compared to recovery period on the basal diet (Nat group). However, neither the antibiotic treatment nor the probiotic treatment had any effects on the average egg weight (AEW), average daily feed intake (AFDI), and feather condition score of laying hens (*P* > 0.05).

**TABLE 3:** Effect of different dietary treatments on the production performance of laying hens.

Group	Con	Ant	*P*-value	Nat	Pro	*P*-value
Egg production (%)	74.34 ± 3.78^a^	71.29 ± 2.84^b^	0.037	67.52 ± 5.82^b^	71.55 ± 3.19^a^	0.048
AEW (g)	62.42 ± 0.86	62.31 ± 0.93	0.761	63.63 ± 2.13	63.26 ± 1.20	0.665
AFDI (g)	122.47 ± 6.47	123.38 ± 3.17	0.673	129.95 ± 4.48	130.67 ± 7.25	0.774
FCR	2.65 ± 0.20	2.85 ± 0.23	0.086	3.08 ± 0.26	2.91 ± 0.21	0.109
Average feather score	2.49 ± 0.38	2.35 ± 0.56	0.309	2.38 ± 0.45	2.21 ± 0.62	0.190

^a,b^*P* < 0.05, and Means within a line followed by different superscript letters differ significantly.

### Behavior

To assess whether chronic antibiotics treatment induces SFP behavior in laying hens and if probiotic (*Lactobacillus rhamnosus LR-32*) supplementation can resolve antibiotic-induced SFP or shift the behavior to GFP, which represents a positive interactive behavior, we evaluated the video recording of hens over 2 weeks. As expected, antibiotic-treated birds showed more tendency for SFP behavior than the control birds. Similar to the effect on egg production, probiotic treatment significantly reversed the effect of antibiotic, with hens in the Pro group displaying significantly less SFP behavior relative to the Nat group hens. Moreover, the frequency of SFP was nearly one-fold lower in probiotic-treated hens than in the antibiotic-treated hens (Ant vs. Pro; 0.69 ± 0.43 vs. 0.34 ± 0.25). On average, 23.5% of the antibiotic-treated birds are classified as severe feather peckers, while only 12.5% of the probiotic-treated birds are in this category. No difference was noted in GFP tendencies between hens treated with either antibiotics or probiotics (*P* > 0.05; Table 4).

**TABLE 4:** Effect of different dietary treatments on feather-pecking behavior in laying hens.

Behavior	Treatment	Class	Chi-square statistic	*P*-value	^1^Frequency (N/min)
GFP	Period of flora disorders	Con	*X*^2^ ^(1, *N* = 648)^ = 0.334	0.563	0.87 ± 0.50
		Ant			1.04 ± 0.32
	Period of flora recovery	Nat	*X*^2^_(1, *N* = 0648)_ = 0.688	0.407	0.94 ± 0.57
		Pro			1.25 ± 0.37
SFP	Period of flora disorders	Con	*X*^2^_(1, *N* = 648)_ = 5.641	0.018	0.12 ± 0.17
		Ant			0.69 ± 0.43
	Period of flora recovery	Nat	*X*^2^_(1, *N* = 648)_ = 4.172	0.041	0.59 ± 0.28
		Pro			0.34 ± 0.25

^1^Behavioral frequencies were calculated from hens randomly selected for sampling (*n* = 7) and are only descriptive statistics.

### Gut microbial composition

**FIGURE 2:**
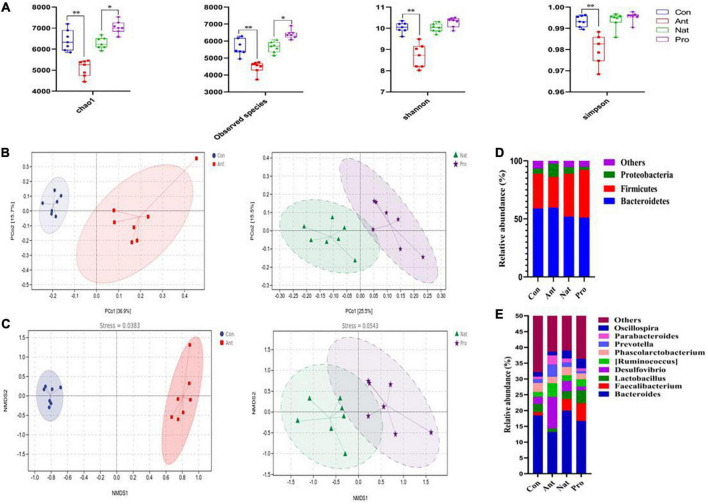
Effects of dietary probiotic supplementation on the gut microbial diversity of antibiotic-treated hens. **(A)** Analysis of the alpha diversity of the gut microbiota by Chao-1, Observed species, and Shannon and Simpson indexes. Differences are assessed by the Mann–Whitney U test with significance defined as *P < 0.05, **P < 0.01. **(B)** Principal coordinates analysis (PCoA) plots were generated using OTU metrics based on the weighted UniFrac distance similarity for the samples in different groups highlighted with different colors. **(C)** Non-metric multidimensional scaling (NMDS) at the genus level. Points of different colors or shapes represent samples in different treatment groups. **(D)** Graph showing the mean percentage of the main phylum level of the cecal microbiota (*n* = 7). **(E)** Graph showing the mean percentage of the main genus level of the cecal microbiota (*n* = 7).

To determine the effect of antibiotic and probiotic treatments on the gut microbiota of the hens, we performed 16S rRNA sequencing of cecum samples and analyzed the abundance of microbial operational taxonomy units. Alpha diversity analysis by Chao1, Observed species, Shannon and Simpson indexes showed that antibiotic treatment significantly reduced the values of all indexes compared to control (all *P* < 0.01; Figure 2A). Probiotic treatment significantly increased the Chao1 (*P* = 0.018) and observed species (*P* = 0.025) indexes compared to the Nat group. Although the values of the Shannon and the Simpson indexes for the Pro group are numerically higher than that of the Nat group, the difference is not statistically significant (*P* = 0.11 and 0.65, respectively).

Beta diversity was further evaluated with weighted UniFrac distance-based principal coordinates analysis (PCoA) at the OTU level (Figure 2B). The PCoA plot showed a clear separation between the Con and Ant groups along the first principal coordinates (36.9% of overall variation) with statistical significance (*F* = 5.866, *P* = 0.001). Also, significant differences in OTU levels were observed between Pro group and Nat group (*F* = 2.235, *P* = 0.012), although the Pro group cluster was not completely separated from the Nat group. Further analysis using non-metric multidimensional scaling analysis (NMDS) demonstrated that the genus level of the microbial communities was also significantly divided into 2 groups by antibiotic or probiotic treatment (Figure 2C). Analyzing the bacterial populations at the phylum level showed that the cecum of Con birds was dominated by *Bacteroidetes* (58.91%; Figure 2D), *Firmicutes* (29.72%), *Proteobacteria* (4.56%), and minority populations (6.81%) such as *Actinobacteria* (0.84%), *Synergistetes* (0.62%), and *Fusobacteria* (0.11%). Antibiotic treatment significantly expanded the *Proteobacteria* (11.40%, *P* < 0.001; Figure 2D) at the expense of *Firmicutes* (26.43%, *P* < 0.05) and others (2.27%, *P* < 0.001). Interestingly, we found that probiotic treatment significantly reduced the abundance of *Proteobacteria* (5.68% VS. 2.38%, *P* < 0.001; Figure 2D), while increasing the abundance of *Firmicutes* (37.14% VS. 41.17%, *P*<0.05) compared to the Nat group hens. At the genus level, the *Bacteroides*, *Faecalibacterium*, *Lactobacillus*, *Desulfovibrio*, *Ruminococcus*, *Phascolarctobacterium*, *Prevotella*, *Parabacteroides*, and *Oscillospira* accounted for the largest proportion of the microbiota (Relative abundance >1%, Figure 2E). The abundance of *Desulfovibrio* (2.31% VS. 10.01%, *P* < 0.01) was increased in antibiotic challenged birds but diminished by *Lactobacillus rhamnosus LR-32* addition (3.25% VS. 1.22%, *P* < 0.01), which also induced an increase in *Faecalibacterium* (3.73% VS. 5.63%, *P* < 0.05) and *Lactobacillus* (2.44% VS. 4.07%, *P* < 0.05) abundance compared to the Nat group hens.

**FIGURE 3:**
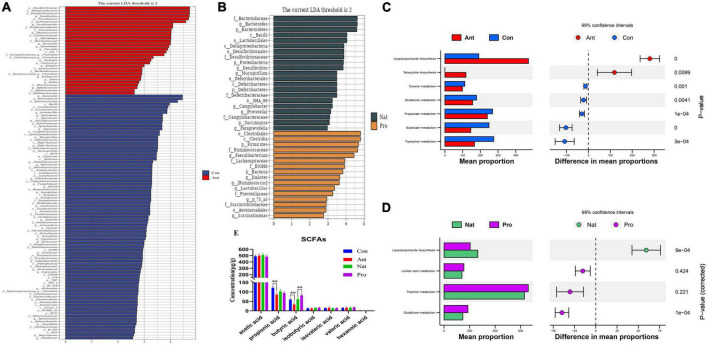
Effects of dietary probiotic supplementation on functions of the gut microbial communities in antibiotic-treated hens. **(A)** Linear discriminant analysis (LDA) comparing the effect size of taxa between Con (blue, *n* = 7) and Ant (red, *n* = 7) birds. Bar plot showing the significant differentially expressed taxa based on effect size [LDA score (log 10) > 2]. **(B)** Linear discriminant analysis (LDA) comparing the effect size of taxa between Nat (brown, *n* = 7) and Pro (yellow, *n* = 7) birds. Bar plot showing the significant differentially expressed taxa based on effect size [LDA score (log 10) > 2]. **(C)** Functional profiling by PICRUSt and Kyoto Encyclopedia of Genes and Genomes (KEGG) categories between Con and Ant birds. Bar graph (left) showing the most significant functional pathways and whisker plot (right) showing their respective significance level. **(D)** Functional profiling by PICRUSt and KEGG categories between Nat and Pro birds. Bar graph (left) showing the most significant functional pathways and whisker plot (right) showing their respective significance level. **(E)** Changes in short-chain fatty acids (SCFAs) concentrations in laying hens after antibiotic and probiotic treatments. Data presented as mean ± SD with significance defined as **P < 0.01.

To explore the bacterial groups that may contribute to the differences in gut microbiota diversity after antibiotic or probiotic treatment, we identified differentially abundant bacterial categories via cladogram generated by LEfSE analyses. A total of 74 bacterial taxa changed significantly between the hen control and antibiotic-treated groups (Figure 3A). The genera that are increased in abundance in the Ant group include *Desulfovibrio*, *Parabacteroides*, *Ruminococcus, Algoriphagus, Barnesiella, Xenorhabdus*, and *Prevotella*, while the abundance of *Bacteroides*, *Lactobacillus*, *Faecalibacterium, Treponema*, and *Mucispirillum* were reduced relative to the Con group hens. On the other hand, A total of 37 bacterial taxa changed significantly between the hen Nat and probiotic-treated groups (Figure 3B). The abundance of *Desulfovibrio and Prevotella* in the Nat group remained at a high level, whereas the probiotic-treated hens show an increased abundance of *Faecalibacterium*, *Lactobacillus, Ruminococcus*, *Succinatimonas*, and *Dialister*.

Next, PICRUSt was used to predict the biological processes that may be relevant to these bacterial communities. KEGG pathway analysis identified significant changes in functions relating to lipopolysaccharide biosynthesis, tetracycline biosynthesis, tyrosine metabolism, glutathione metabolism, propanoate metabolism, butanoate metabolism and tryptophan metabolism with antibiotic treatment (Figure 3C). These functions are also partially altered by the probiotic treatment (Figure 3D).

Alternation in the gut microbiota can impact propanoate and butanoate metabolism, leading to changes in intestinal metabolites. To determine how antibiotic and probiotic treatments may affect intestinal metabolites, we evaluated the levels of short-chain fatty acids (SCFAs) in cecum samples by liquid chromatography tandem mass spectrometry (LC-MS/MS). Antibiotic treatment significantly decreased the levels of propionic acid and butyric acid in the cecum of laying hens compared to control (Figure 3E). In contrast, probiotic treatment enhanced the level of butyric acid in the cecum of laying hens (Figure 3E).

### Gut barrier

**FIGURE 4:**
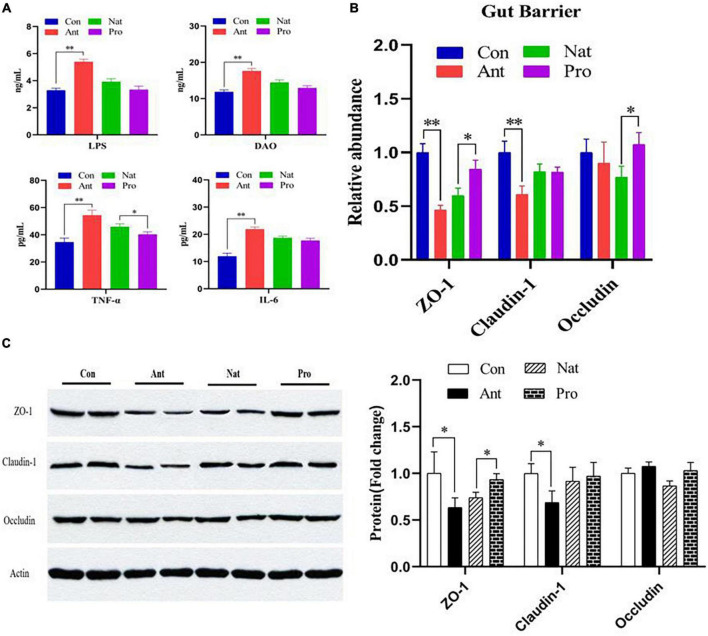
Effects of dietary probiotic supplementation on the ileum barrier integrity and inflammation response in antibiotic-treated hens. **(A)** Graphs showing the concentrations of LPS, DAO, TNF-α, and IL-6 in the serum of laying hens following different treatments. **(B)** Graph showing the relative mRNA expression of ZO-1, claudin-1, and occludin in the ileum of laying hen (*n* = 7). The β-actin was used as an internal control. **(C)** Representative Western blot (left) and quantified protein expression data (right) of ZO-1, claudin-1, and occludin ileum the ileum of laying hen (*n* = 4). Quantification data were normalized to β-actin. Data presented as mean ± SD with significance defined as *P < 0.05, **P < 0.01.

The LPS biosynthesis pathway is significantly altered in the gut microbial function analysis above, prompting us to evaluate the levels of LPS in the serum. Consistent with the high abundance of lipopolysaccharide biosynthesis pathways in the KEGG analysis, serum LPS concentrations were significantly higher in the antibiotic-treated hens compared to controls (*P* < 0.001, Figure 4A). In addition, inflammatory cytokines such as TNF-α and IL-6 were also increased by antibiotic treatment. All three proteins are reduced in the Nat group and probiotic treatment did not show additional effect, except for TNF-α where significant reduction is observed (*P* = 0.027). Intestinal barrier permeability was then examined by evaluating diamine oxidase (DAO), a biomarker of barrier damage. Serum DAO level was elevated in the Ant group relative to the Con group but probiotic treatment did not show a significant effect compared to natural recovery (Figure 4A). Antibiotic treatment significantly reduced the expression of tight junction proteins, ZO-1 and Claudin-1, but not occludin, in the ileum of laying hens at both the mRNA (Figure 4B) and protein level (Figure 4C), indicating that antibiotic treatment impairs the permeability of the intestinal barrier. Probiotic treatment markedly upregulated the relative mRNA and protein abundance of ZO-1 (*P* = 0.012, Figure 4B) and the mRNA levels of occludin (*P* = 0.023).

### Blood-brain barrier

**FIGURE 5:**
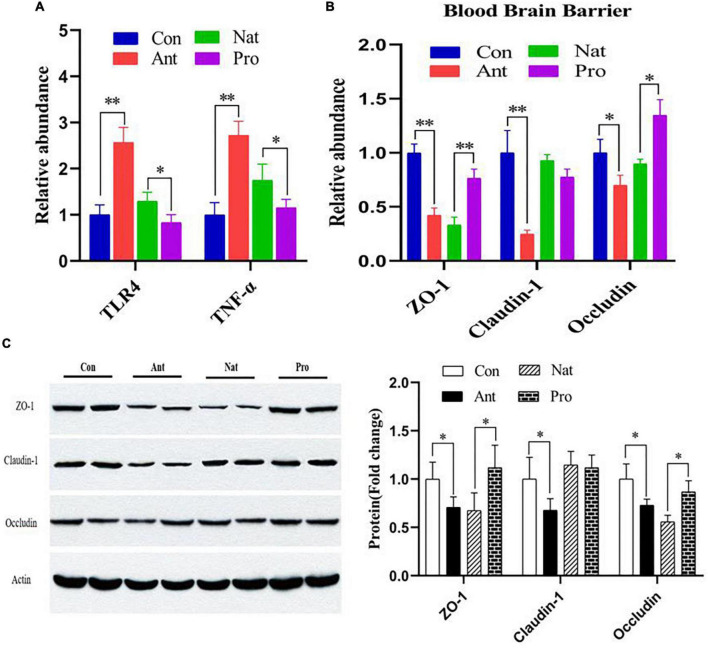
Effects of dietary probiotic supplementation on the hypothalamus barrier integrity and inflammation response in the hypothalamus of antibiotic-treated hens. **(A)** Graph showing the relative mRNA expression of TLR4 and TNF-α in the hypothalamus of laying hens. **(B)** Graph showing the mRNA expression of ZO-1, Claudin-1, and Occludin in the hypothalamus of laying hen (*n* = 7) following different treatments normalized to β-actin. **(C)** Representative Western blot (left) and quantified expression data (right) of ZO-1, claudin-1, and occludin in the hypothalamus of laying hen (*n* = 4). Data presented as mean ± SD with significance defined as *P < 0.05, **P < 0.01.

We next evaluated effect of antibiotic and probiotic treatments on brain inflammation and the integrity of the blood-brain barrier (BBB) in the hypothalamus of laying hens. The results reveal that antibiotic treatment significantly increased the gene expression levels of TLR4 and TNF-α (Figure 5A), while probiotic treatments significantly reduced their expression compared to the Nat group (both *P* < 0.001). Similar to the observation in the ileum, hypothalamic levels of ZO-1 and claudin-1 mRNA (Figure 5B), proteins (Figure 5C) are significantly reduced by antibiotic treatment. Probiotics significantly restored the expression of ZO-1 but not claudin-1. Unlike the ileum observation, occludin expression in the hypothalamus is significantly reduced at the gene and protein levels by antibiotic treatment, which can again be rescued by probiotic treatment. These results indicate that the restorative effects of probiotics on the brain may involve inhibiting the antibiotic-induced increase in permeability of the gut-brain barrier and inflammatory responses.

### Serotonin signaling

**FIGURE 6:**
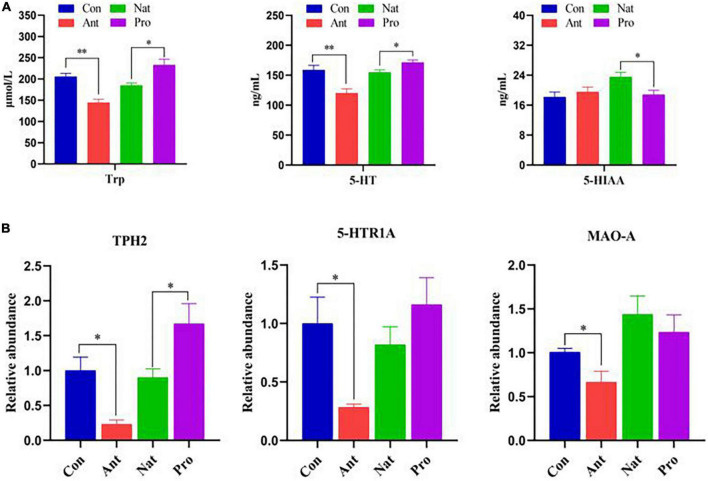
Effects of dietary probiotic supplementation on the levels of 5-HT metabolism-related proteins in the hypothalamus and serum of antibiotic-treated hens. **(A)** Graph showing the concentration of Try, 5-HT, and 5-HIAA in the serum of laying hens (*n* = 7). **(B)** Graph showing the relative mRNA levels of TPH-2, 5-HTRl A and MAO-A in the hypothalamus of laying hens (*n* = 7). Data were normalized to β-actin and the significance defined as *P < 0.05, **P < 0.01.

We further examined the effect of antibiotic treatment on 5-HT and its metabolites and the potential positive effect of probiotic treatment on 5-HT metabolism in laying hens. Compared to the Con group, antibiotic treatment significantly decreased the serum concentrations of tryptophan (Trp), a precursor for the synthesis of 5-HT, and 5-HT itself, but did not affect the level of its metabolite 5-HIAA (Figure 6A). Probiotic treatment increased the concentration of Trp and 5-HT but inhibited 5-HIAA compared to the Nat group. In addition, antibiotic treatment significantly reduced the hypothalamic mRNA levels of tryptophan hydroxylase (TPH2), the 5-HT receptor 5-HTR1A, and monoamine oxidase A (MAO-A; Figure 6B). Probiotic treatment significantly increased TPH2 levels; the slight increase in 5-HTR1A and reduction in MAO-A was not statistically significant.

### Probiotic-altered gut microbiota correlates with physiological indicators and behavior in laying hens

**FIGURE 7:**
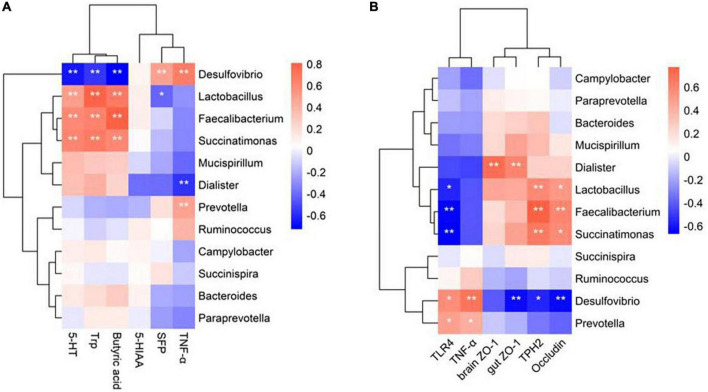
Spearman’s correlations between the gut microbiota and the physiological indicators. **(A)** Heatmap of correlation coefficients between the intestinal microbial phylotypes of significant differences and SFP behavior, intestinal metabolite of butyric acid, 5-HT metabolism and TNF-α in the serum. **(B)** Heatmap of correlation coefficients between the intestinal microbial phylotypes of significant differences and Inflammation-related genes expression, and tight junction-related genes expression in the gut and brain. Red represents a positive correlation, and blue indicates negative correlations. *P < 0.05, **P < 0.01.

A Spearman correlation analysis was conducted on the top 12 differential bacteria, and SFP, as well as altered physiological indicators such as serum 5-HT metabolism, TNF-α levels, an intestinal metabolite of butyric acid, and gene expression of gut-brain barrier function in laying hens to explore correlations between altered gut microbiota due to probiotic treatment and changes in behavioral and physiological indicators (Figure 7). Overall, changes in behavior and physiology were significantly associated with six main differential genera of bacteria. Probiotic-enhanced bacteria were positively correlated, and probiotic-reduced bacteria were negatively correlated with tight junction-related gene expression, 5-HT metabolite-related pathways, and butyric acid levels. Among the genera, *Faecalibacterium*, *Lactobacillus*, and *Succinatimonas* exhibited a positive correlation with the concentration of Trp and 5-HT in the serum (Figure 7A), and gene expression of TPH2 and Occludin (Figure 7B). The abundance of *Desulfovibrio and Prevotella* were positively correlated with SFP behavior, and the serum TNF-α concentration, and gene expression of TLR4 and TNF-α in the brain (Figure 7B). *Dialister* was positively related to gene expression of ZO-1 in the gut and brain (Figure 7B). Conversely, the abundance of *Desulfovibrio* had a negative correlation with serum concentration of 5-HT and Trp and butyric acid (Figure 7A), and gene expression of TPH2, ZO-1, and occludin (Figure 7B). Increased abundance of *Lactobacillus* was associated with the decreased SFP behavior (Figure 7A). Thus, *Desulfovibrio*, *Prevotella*, *Faecalibacterium, Lactobacillus and Succinatimonas, and Dialister* might be the key bacteria for probiotic treatment to reduce SFP behavior in laying hens; these collectively might be altering 5-HT metabolism, inflammatory response and barrier function at the gut-brain axis.

## Discussion

In the current study, we found that the impacts of chronic lincomycin hydrochloride exposure on egg production and FP behavior in laying hens are associated with changes in the microbial-gut-brain axis, including reduced diversity of gut microbial communities and production of SCFAs, disruption of the gut barrier and blood-brain barrier proteins, increased inflammation, and inhibition of 5-HT metabolic pathways. In contrast, dietary supplementation with a single *Lactobacillus rhamnosus* strain mitigated the negative effects of antibiotic on performance and behavior in laying hens by reversing the changes in the gut-brain axis. Thus, our data provide insights into the link between microbiota and FP behavior in laying hens and a potential role for probiotics in regulating the brain-gut axis.

Increasing evidence suggests that changes in the gut microbiota affect physiological and behavioral processes directly relating to wellbeing, such as changes in stress, anxiety, social behavior, and memory (Sampson and Mazmanian, 2015). As the most direct method of disrupting the gut microbial ecology (Champagne-Jorgensen et al., 2019), antibiotic treatment can cause mice to exhibit anxiety-like behaviors and higher levels of aggression in several tests, while supplementation with *Lactobacillus rhamnosus JB-1* prevented some of these changes (Leclercq et al., 2017). Feather-pecking behavior in hens is considered an example of anxiety behavior (Mott et al., 2022) and has been found to increase in frequency with chronic exposure to antibiotics in this study. However, our study demonstrated that administering *Lactobacillus rhamnosus LR-32* could reduce SFP behavior following antibiotic exposure. This is consistent with previous research by Mindus et al. (2021), who found that *Lactobacillus* supplementation can prevent SFP behavior in adult laying hens by restoring low levels of *Lactobacillus* reported in hens with this behavior (Birkl et al., 2018; van der Eijk et al., 2019a). Additionally, our population and LEfSe analysis showed that antibiotic treatment can significantly affect the gut microbial composition of hens including increases in the *Proteobacteria* phylum and *Desulfovibrio* genus. *Proteobacteria* are known to be opportunistic pathogens associated with inflammation and anxiety/depression (Jiang et al., 2015; Rychlik, 2020). Specifically, the abundance of *Desulfovibrio* have reported to be positively related to regressive autism and anxiety-like behavior (Zhu et al., 2019). Although our study showed a positive correlation between the abundance of *Desulfovibrio* and severe feather-pecking behavior, further research is needed regarding its specific regulatory mechanism. Interestingly, we also observed a substantial increase in beneficial bacteria such as the *Lactobacillus* and *Faecalibacterium* genus in cecum samples of laying hens in the probiotic group compared to the Nat group. *Lactobacillus* is known to have beneficial effects on the health of poultry when used as a probiotic supplement in poultry diets (Khan et al., 2020). The increased abundance of *Faecalibacterium* in the gut of laying hens challenged with *Salmonella Typhimurium* that turned negative for *Salmonella* (Khan and Chousalkar, 2020), suggesting its potential role as a probiotic candidate for intestinal health. Besides, the caeca digesta and mucosa of the LFP line were more abundant *Faecalibacterium* and *Lactobacillus* compared to the HFP line (Borda-Molina et al., 2021). It is well known that genetic and environmental factors determine the feather pecking behavior of laying hens (Sedlačková et al., 2004), but our results suggest that the gut microbiota are also closely related to feather-pecking behavior, and *Lactobacillus rhamnosus LR-32* can alleviate antibiotic-induced SFP behavior by modulating the gut microbiota.

Based on functional profiling of the microbiota communities induced by antibiotic or probiotic treatment (KEGG pathway analysis) in our study, we verified the effect of different dietary treatments on short-chain fatty acids (SCFAs) intestinal metabolites. We found that *Lactobacillus rhamnosus LR-32* reversed the decrease in the cecum metabolite of butyric acid concentrations in laying hens induced by antibiotic treatment. Previous studies have indicated that intestinal metabolites, such as butyrate and lactate, may contribute to developing feather-pecking behavior (Meyer et al., 2013; Mens et al., 2020). While there is no direct evidence linking butyrate with feather-pecking behavior through blood transmission to the brain in laying hens, emerging evidence suggests that butyrate may regulate the microbiota-gut-brain crosstalk indirectly. Examples include inhibition of intestinal pathogen adhesion (Argañaraz-Martínez et al., 2013), maintenance of intestinal barrier and blood-brain barrier integrity (Li et al., 2016; Yosi et al., 2022), prevention of inflammation (Miao et al., 2022), and direct neuroactive properties (Sullivan, 2020; Liu et al., 2021). The current study demonstrated that serum DAO levels were elevated in laying hen streated with antibiotics, indicating that antibiotic exposure disrupts intestinal barrier function. Blood DAO activity is a marker of intestinal barrier function and intestinal permeability (Nieto et al., 2000). This finding is supported by the decreased expression of tight junction genes and proteins in the ileum of antibiotic-treated hens. Tight junctions play a crucial role in maintaining the integrity of the intestinal barrier (Anderson, 2001). Both natural recovery and *Lactobacillus rhamnosus LR-32* treatment reversed the effect of antibiotics on DAO comparably, suggesting that LR-32 may not be the critical factor in determining DAO expression. On the other hand, *Lactobacillus rhamnosus LR-32* significantly increased the gene and protein levels of ZO-1 in the ileum of laying hens. This is in agreement with the findings of others, where *Lactobacillus* has been shown to remodel the intestinal flora, leading to reversals of antibiotic-induced disruptions of intestinal barrier functions (Gao et al., 2017; Wang et al., 2018; Zhang et al., 2021; Chen et al., 2022; Dahiya and Nigam, 2023). This may be attributed to the increase of butyrate-producing bacteria, such as *Faecalibacterium and Lactobacillus*, after probiotic treatment, which promoted intestinal butyrate secretion. Butyrate not only provides energy substrate for intestinal cells, but also promotes the relative expression of ZO-1 mRNA in intestinal epithelial cells, enhancing transmembrane resistance of epithelial cells and protecting the integrity of the intestinal barrier (Xiao et al., 2023).

Previously, loss of intestinal epithelial cell integrity due to dysbiosis of the gut microbiota have been reported to increase gut-derived bacterial translocation, and allow LPS and other toxic substances to enter the circulation and trigger systemic inflammation, leading to damage to various organs, including the brain (Xi et al., 2019; Fock and Parnova, 2023). LPS is a known ligand of toll-like receptor 4 (TLR4) and induces inflammatory responses by activating the TLR4/nuclear factor κB (NF-κB) signaling pathway (Lu et al., 2008). The results of this study demonstrated that antibiotic treatment led to elevating the levels of LPS and pro-inflammatory factors such as TNF-α and IL-6 in the serum. In addition, TLR4 and TNF-α genes were upregulated, accompanied by a decrease in tight junction protein genes and proteins in the hypothalamus, possibly due to the fact that LPS as an endotoxin can enter the circulation and alter microvascular homeostasis and blood-brain barrier permeability, resulting in disorders of tight junctions and stimulating the production of pro-inflammatory cytokine (Blanchette and Daneman, 2015). Consistent with the previously reported immunomodulatory effects of *Lactobacillus* (Xu et al., 2020; Sureshkumar et al., 2021), we found that antibiotic-induced increases in inflammatory factors such as TNF-α in the blood and hypothalamus, and TLR4 in the hypothalamus were attenuated by *Lactobacillus rhamnosus LR-32* administration, while increasing hypothalamic ZO-1 and occludin gene and protein levels, indicating that *Lactobacillus rhamnosus LR-32* was able to reduce the antibiotic-induced inflammatory response in the brain and the increase in BBB permeability. Long-term effects of antibiotic use early in life have been reported in mouse offspring, who exhibit abnormal social behavior and aggression, and a decrease in the relative levels of SCFA-producing genera in the gut has been observed (Sun et al., 2021), accompanied by an increase in brain levels of pro-inflammatory cytokines, which results in decreased expression of brain occludin and ZO-1 mRNA and increased BBB permeability. In contrast, these behavior alterations were prevented by supplementation with *Lactobacillus rhamnosus*, a SCFA producer, and restoration of tight junction proteins and BBB permeability in the hippocampal region was observed (Wen et al., 2020). As we discussed above, we hypothesize that *Lactobacillus rhamnosus LR-32* may have a similar mechanism to reduce abnormal behavior by restoring intestinal microbiota and gut-brain barrier function. This is supported by our results showing that probiotic-enhanced bacteria were negatively correlated with SFP behavior and TLR4 gene expression and positively correlated with intestinal butyric acid content and tight junction-related gene expression, while probiotic-reduced bacteria were positively correlated with SFP behavior and inflammation-related gene expression.

Of note, *Lactobacillus* has been shown to have a direct, potentially active effect on neurotransmission within the gut-brain axis, by recruiting tryptophan or limiting its availability to the host; potentially altering the 5-HT metabolic pathway of the gut-brain axis to affect animal behavior (Carrero, 2021). The importance of the central serotonergic system in modulating feather-pecking behavior is, indeed, suggested by several studies in avian species (Flisikowski et al., 2009; Kops et al., 2013). However, as it is impossible to determine serotonergic signaling activities in the brain in a cell-specific manner, we measured the expression of metabolic genes related to 5-HT signaling. In the brain, the down-regulation of 5-HTR1A, and TPH2, and MAO-A indicated an inhibition of 5-HT synthesis, release and metabolic processes, respectively (Chenxuan et al., 2021). Our data confirm that 5-HT biosynthesis and catabolism were inhibited after antibiotic treatment, whereas dietary supplementation with *Lactobacillus rhamnosus LR-32* significantly upregulated TPH2 gene expression and enhanced central 5-HT synthesis. In addition, a positive correlation has been drawn between blood 5-HT and central 5-HT concentrations in the brain (Uitdehaag et al., 2011; Yubero-Lahoz et al., 2014). This corresponds to our results of changes in serum Trp and 5-HT concentrations. The correlation analysis also revealed a potential regulatory role of gut microorganisms including *Faecalibacterium*, *Lactobacillus*, and *Succinatimonas* on 5-HT metabolism in laying hens. Although the role of *Lactobacillus rhamnosus LR-32* in restoring lincomycin hydrochloride mediated reduction of 5-HT in the gut-brain axis may contribute to the alleviation of feather-pecking behavior in laying hens, the detailed mechanism remains to be further investigated.

## Conclusion

The restorative effects of *Lactobacillus rhamnosus LR-32* supplementation on hens challenged with lincomycin hydrochloride resulted from improvements in gut dysbiosis, butyric acid production, gut-brain barrier function, and 5-HT metabolism. These activities slowed the decline in egg production and reduced SFP behavior in hens. *Lactobacillus rhamnosus LR-32* can be used as an intervention strategy to reduce feather pecking behavior in laying hens. Although correlation analysis showed that the abundance of *Lactobacillus* and *Desulfovibrio* were closely related to most physiological indicators and severe feather pecking behavior (Figure 7), the mechanisms by which *Lactobacillus rhamnosus LR-32* exerts a restorative effect on SFP behavior by modulating gut microbiota need further investigation.

## Data availability statement

The original contributions presented in this study are included in the article/supplementary material, further inquiries can be directed to the corresponding authors.

## Ethics statement

This animal study was reviewed and approved by the Animal Ethics Committee of Hebei Agricultural University (Permit Number: HB/2021/03).

## Author contributions

CH: investigation, methodology, writing—original draft, and writing—review and editing. QY: investigation, methodology, writing—original draft, and writing—review. LSu and KD: investigation and methodology. DY: investigation and software. EH: visualization and software. DW: methodology. YC: investigation. LSh: resources and project administration. RZ: writing—review and editing. GZ: supervision and project administration. HC: supervision, project administration, and funding acquisition. All authors contributed to the article and approved the submitted version.

## Footnotes

1
http://sourceforge.net/projects/rdp-classifier/

2
http://www.arb-silva.de

3
http://qiime.org/tutorials/tutorial.html
